# Supplementary material for: Structural basis of cyclobutane pyrimidine dimer recognition by UV-DDB in the nucleosome
Source: Nat Commun. 2025 Nov 11;16:9709. doi: 10.1038/s41467-025-65486-5 (PMC12606344; doi:10.1038/s41467-025-65486-5)
Supplement: Supplementary file 1 — Supplementary Information [file 41467_2025_65486_MOESM1_ESM.pdf]

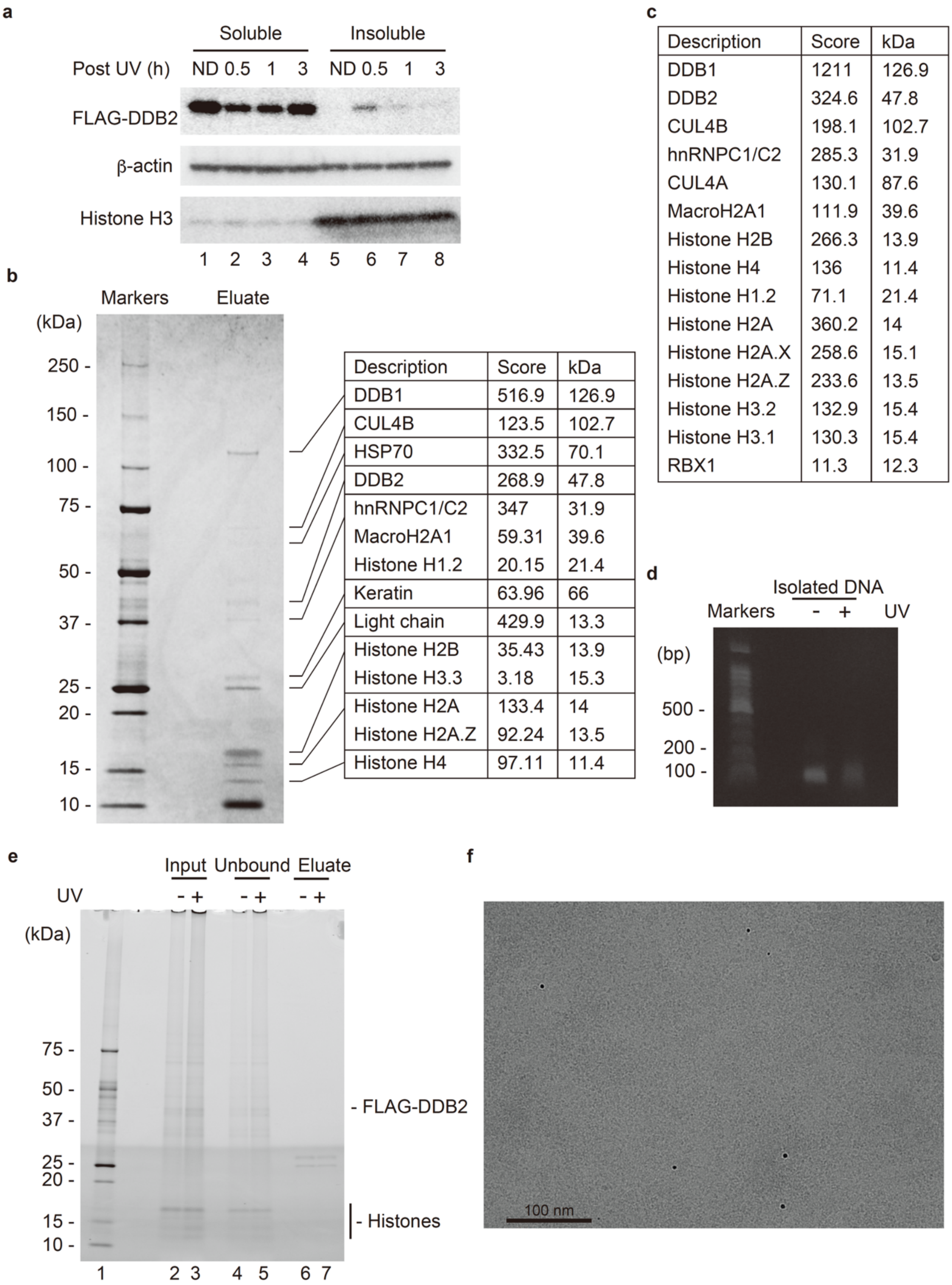

Description	Score	kDa
DDB1	516.9	126.9
CUL4B	123.5	102.7
HSP70	332.5	70.1
DDB2	268.9	47.8
hnRNPC1/C2	347	31.9
MacroH2A1	59.31	39.6
Histone H1.2	20.15	21.4
Keratin	63.96	66
Light chain	429.9	13.3
Histone H2B	35.43	13.9
Histone H3.3	3.18	15.3
Histone H2A	133.4	14
Histone H2A.Z	92.24	13.5
Histone H4	97.11	11.4

Description	Score	kDa
DDB1	1211	126.9
DDB2	324.6	47.8
CUL4B	198.1	102.7
hnRNPC1/C2	285.3	31.9
CUL4A	130.1	87.6
MacroH2A1	111.9	39.6
Histone H2B	266.3	13.9
Histone H4	136	11.4
Histone H1.2	71.1	21.4
Histone H2A	360.2	14
Histone H2A.X	258.6	15.1
Histone H2A.Z	233.6	13.5
Histone H3.2	132.9	15.4
Histone H3.1	130.3	15.4
RBX1	11.3	12.3

Supplementary figures 1: DNA recognition of UV-DDB complex in chromatin

a, FLAG-DDB2 was detected by western blotting, using an anti-DDB2 antibody, an anti-β-actin antibody, and an anti-Histone H3 antibody. **b,** Mass spectrometry analyses were performed with the eluted fraction prepared by the method described in Fig. 1b, but

without crosslinking. HEK293F expressing FLAG-DDB2 was used in this experiment. All detected bands were digested and loaded onto the column. Detected peptides were identified and indicated. **c,** The same mass spectrometry analyses were performed but the eluted fraction was analyzed by the shotgun method, rather than the in-gel digestion method. Representative proteins are shown. **d,** DNA fragments were isolated and purified from the eluted fraction used for the ChIP-CryoEM method. DNA fragments were subjected to 1% agarose gel electrophoresis and detected by SYBR Safe staining. **e**, The same experiment was performed as in Fig. 1b, but in the presence of 20 μM ATPγS. After an incubation with anti-FLAG beads, each fraction was subjected to SDS-PAGE and stained with Oriole. **f,** Representative Cryo-EM micrograph of the eluted fraction.

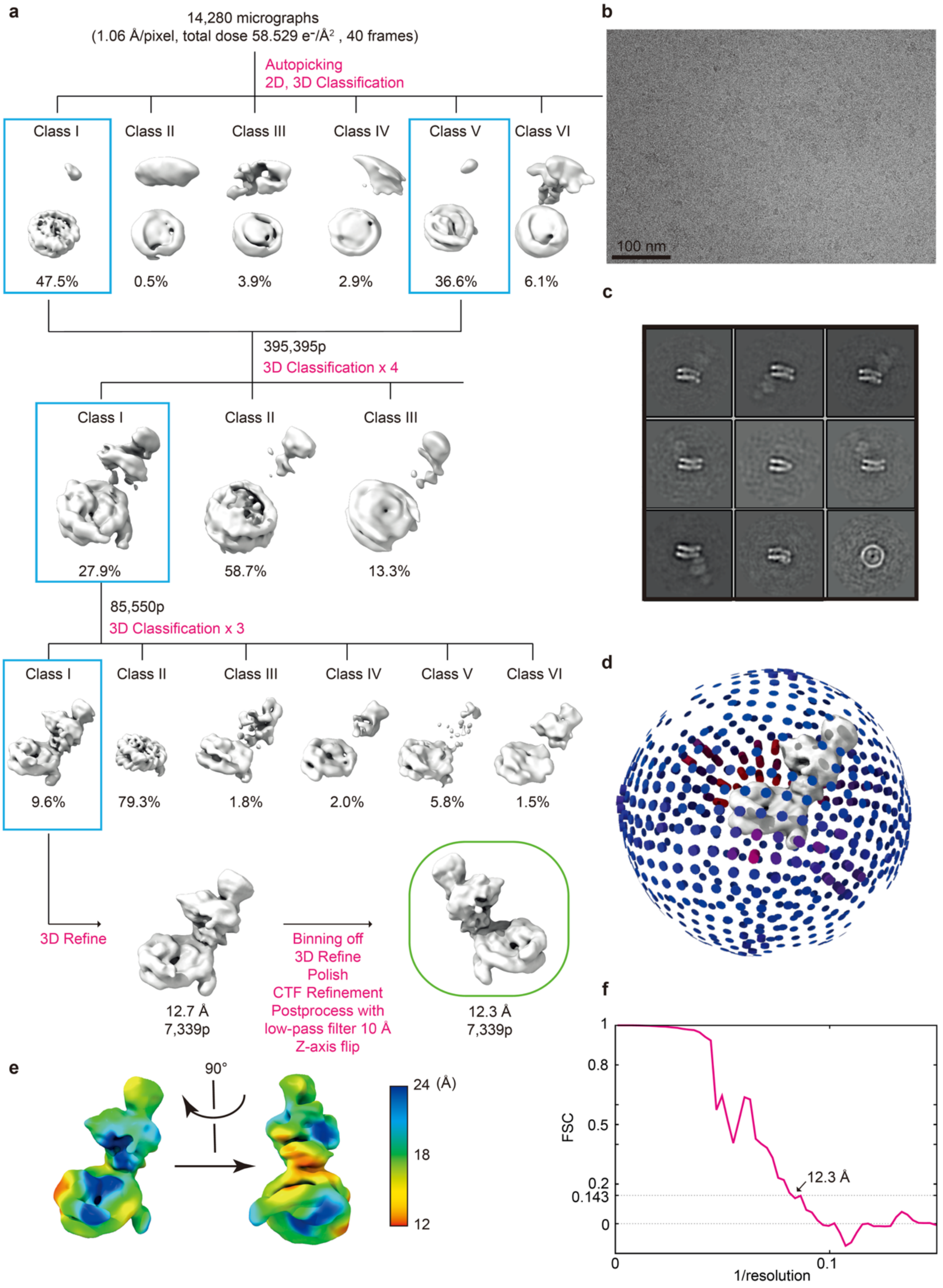

Supplementary figures 2: Cryo-EM analyses of native UV-DDB-NCP complex

a, Classification and refinement procedures for the cryo-EM analyses of the cellular UV-DDB-NCP complex. In total, 14,280 micrographs were captured and further processing

was performed with the Relion 4.0 software. After motion correction, particles were autopicked and extracted. After 2D classification, 6 classes were obtained. Following six rounds of 3D classification, 6 classes were obtained as the final classified model. Class I was refined, and then polishing, CTF refinement, and postprocessing were performed with Relion with a 10 Å low-pass filter. The 12.3 Å resolution map was obtained as the final cryo-EM map. **b,** Representative cryo-EM micrograph. **c,** 2D classification images after initial particle extraction. **d,** Angular distribution of the particles in the final model after 3D refinement. **e,** Local-resolution map of the cellular UV-DDB-NCP complex in the final model, colored by resolution. **f,** Gold-standard Fourier shell correlation curves (FSCs) for the native UV-DDB-NCP complex in the final model.

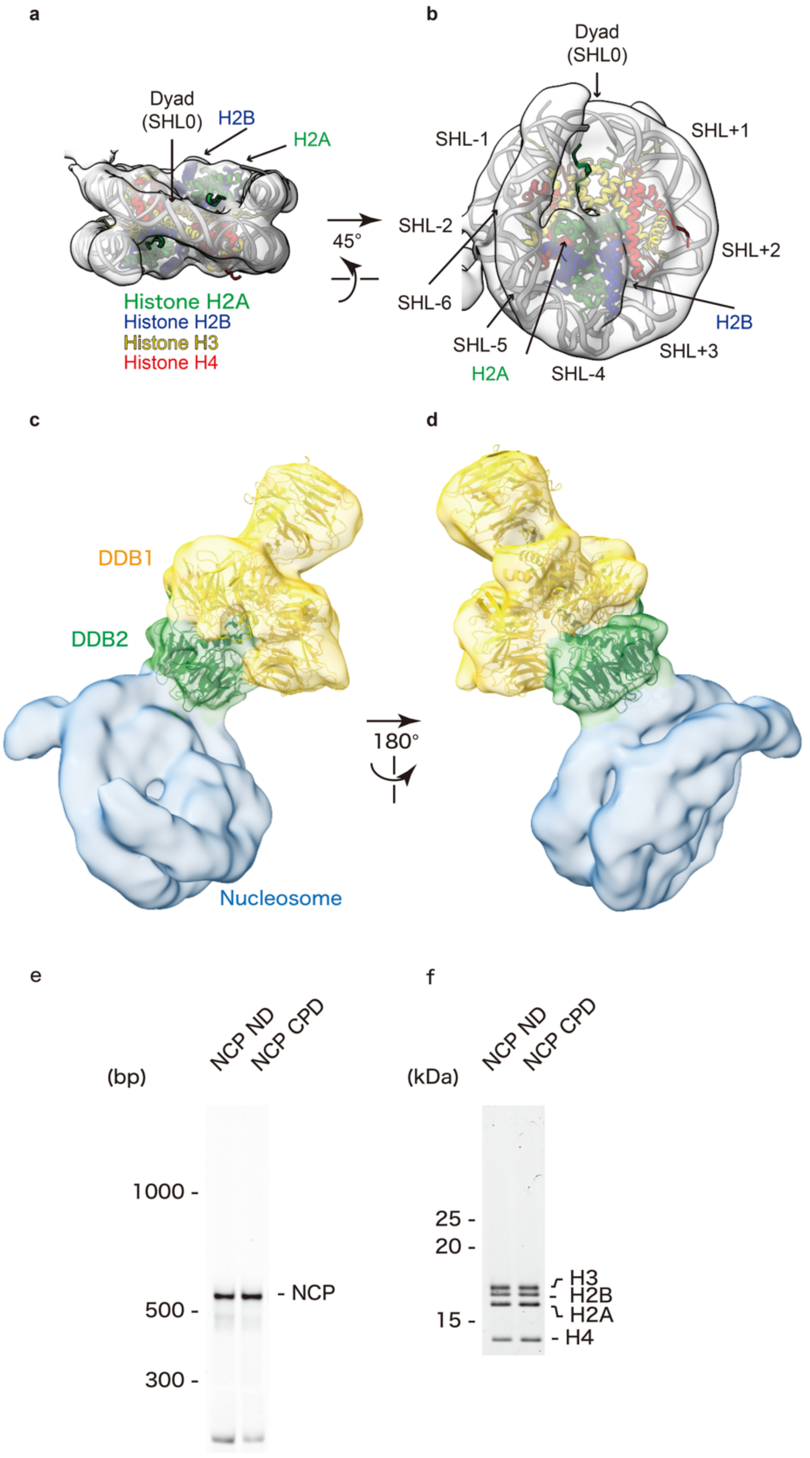

Supplementary figures 3: Cryo-EM analyses of UV-DDB-NCP^{CPD} complex

a, The 3D structural model of the nucleosome (PDB ID: 3AFA) was superimposed onto the cryo-EM map of cellular UV-DDB bound to the nucleosome. **b,** SHLs in the fitted structure are indicated and used to determine the SHL positions in cellular UV-DDB

bound to the nucleosome. **c,d,** The 3D structural model of UV-DDB (PDB ID: 3EI4) was superimposed onto the cryo-EM map of cellular UV-DDB bound to the nucleosome, in two different angles. **e,f,** Reconstituted nucleosomes (NCP ND) or nucleosomes containing CPD (NCP CPD) were subjected to Native-PAGE or SDS-PAGE, and then stained with SYBRGold (**e**) or Oriole (**f**).

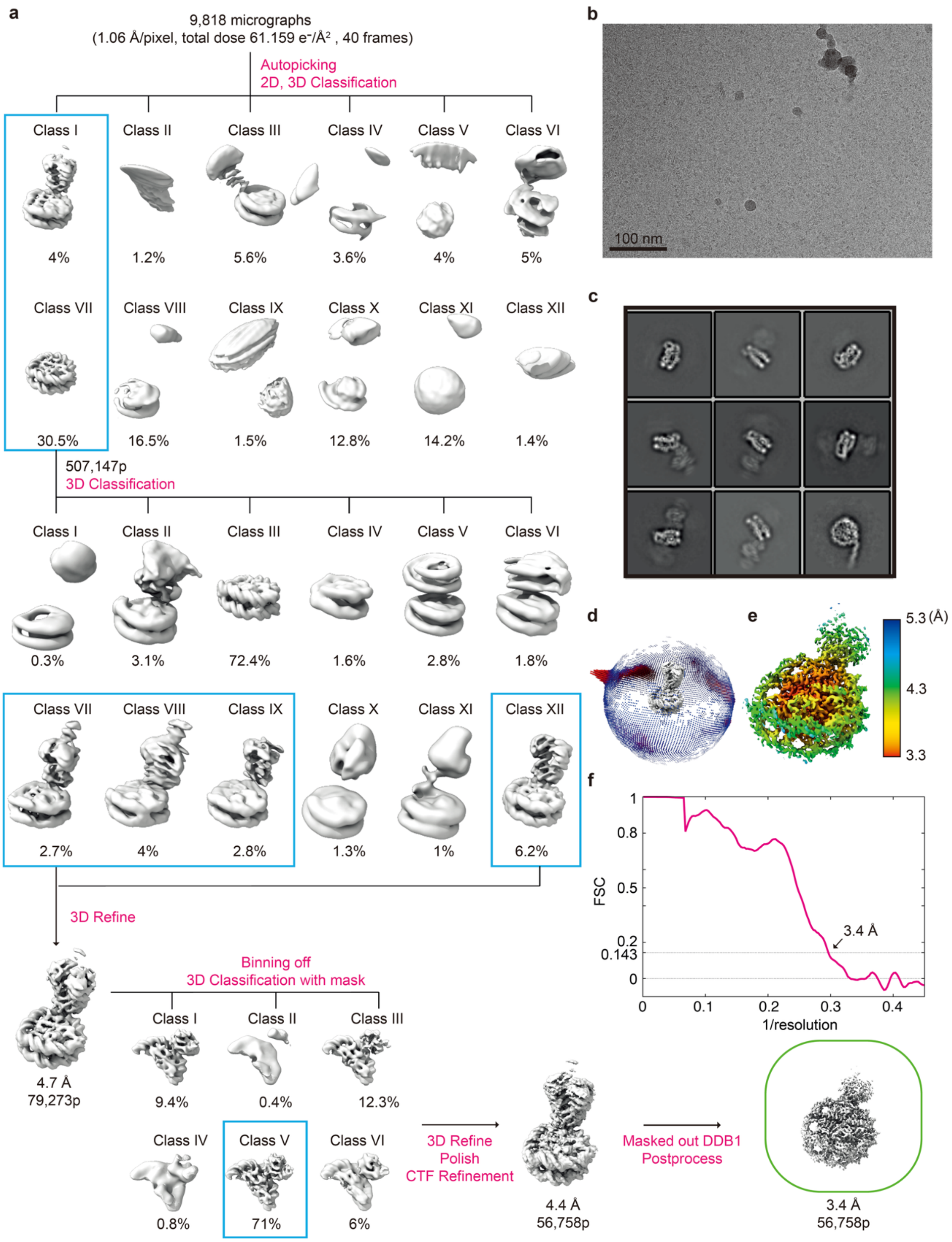

Supplementary figures 4: Cryo-EM analyses of UV-DDB-NCP^{CPD} complex

a, Classification and refinement procedures for the cryo-EM analyses of the UV-DDB-NCP^{CPD} complex. In total, 9,818 micrographs were captured and further processing was performed with the Relion 3.0 software. After motion correction, particles were autopicked and extracted. After 2D classification, 12 classes were obtained. Following

two rounds of 3D classification, 4 classes were obtained and these particles were combined. After 3D refinement, 3D classification was performed using a soft mask on the damaged site. Class V was refined, and then polishing and CTF refinement were performed with Relion. After polishing, the 3.4 Å resolution map was obtained with masking out DDB1. The map sharpened by DeepEMhancer was used as the final cryo-EM map. **b,** Representative cryo-EM micrograph. **c,** 2D classification images after initial particle extraction. **d,** Angular distribution of the particles after the final 3D refinement. **e,** Local-resolution map of the UV-DDB-NCP^{CPD} complex, colored by resolution. **f,** Gold-standard Fourier shell correlation curves (FSCs) for the UV-DDB-NCP^{CPD} complex after polishing.

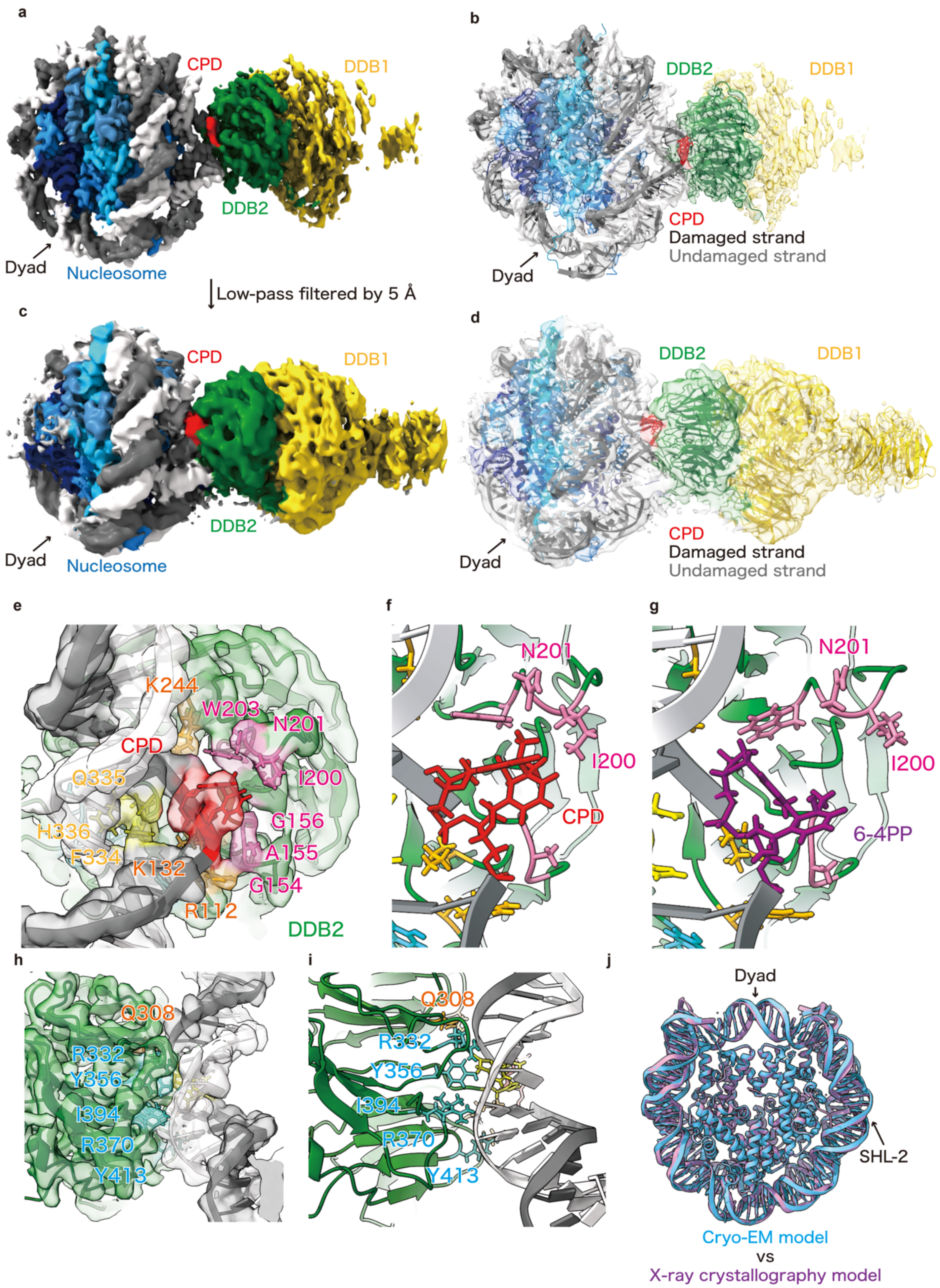

Supplementary figures 5: 3D structural model of the UV-DDB-NCP^{CPD} complex

a, Cryo-EM map of the UV-DDB-NCP^{CPD} complex at 3.4 Å resolution. **b,** The 3D structural model of the UV-DDB-NCP^{CPD} complex (PDB ID: 9J8W) corresponding to the

cryo-EM map was determined by the Phenix software. NCP_THF2(-1)-UV-DDB (PDB ID: 6R8Z) was used as the initial model. **c,** Cryo-EM map of the UV-DDB-NCP^{CPD} complex filtered by a 5 Å low-pass filter to enhance the volume of DDB1. **d,** DDB1 (PDB ID: 6R8Y) was superimposed onto the cryo-EM map of the UV-DDB-NCP^{CPD} complex filtered by a 5 Å low-pass filter. **e-g,** 3D model vs. cryo-EM map for the UV-DDB-NCP^{CPD} complex. Overall structure of the contact surface of DDB2 to CPD (**e**), contact residues of DDB2 to CPD (**f**) or 6-4PP (PDB ID: 6R8Y) (**g**), and contact surface of DDB2 to the DNA backbone (**h**) are indicated. **i,** Cartoon model of the contact surface of DDB2 to the DNA backbone. **j,** Models of the nucleosome with the 601L sequence determined by cryo-EM analysis (PDB ID: 7VZ4) (cyan) and the nucleosome with the α-satellite sequence determined by X-ray crystallographic analysis (PDB ID: 3AFA) (purple) were compared and overlayed. No significant effects of crystal packing were observed.

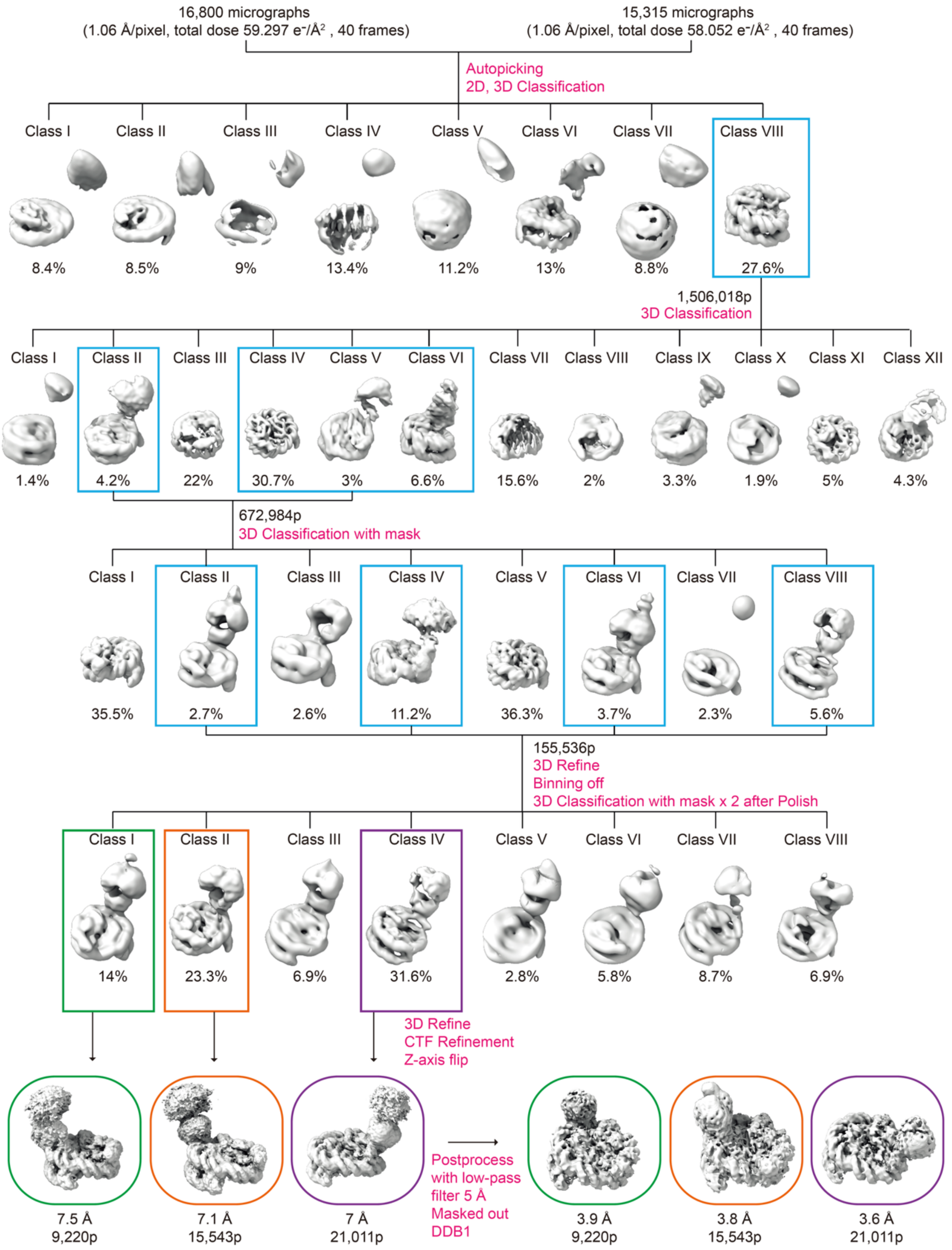

Supplementary figures 6: Procedures for cryo-EM analyses of UV-DDB bound to native nucleosomes

Two different microscopy sessions were performed, and 32,115 micrographs were captured and processed with the Relion 4.0 software. After motion correction, particles were autopicked and extracted. After 2D classification, 8 classes were obtained after 3D

classification. Following two rounds of 3D classification, 4 classes were combined and refined without binning. After an additional two rounds of 3D classification, three different maps were classified. After 3D refinement and CTF refinement, 7.5 Å, 7.1 Å, and 7 Å resolution maps were obtained. Polishing was performed in Relion with a 5 Å low-pass filter masking out DDB1 to reduce flexibility and obtain higher resolution, and the resultant 3.9 Å, 3.8 Å, and 3.6 Å resolution maps were obtained, respectively.

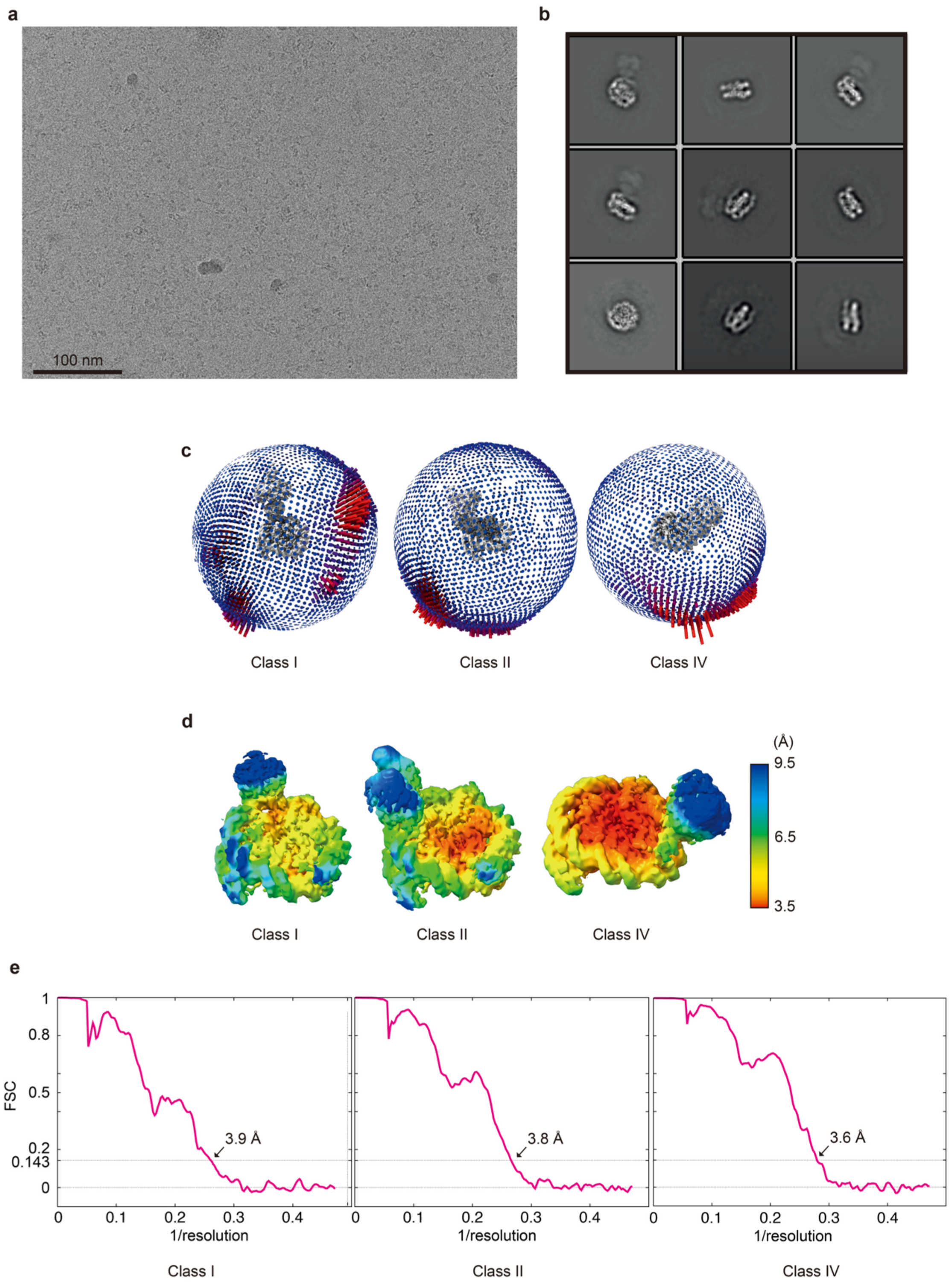

84 **Supplementary figures 7: Cryo-EM analyses of UV-DDB bound to native**
85 **nucleosomes**

86 **a,** Representative cryo-EM micrograph. **b,** 2D classification images after the initial
87 particle extraction. **c,** Angular distributions of the particles after the final 3D refinement.

d, Local-resolution maps of UV-DDB bound to the native NCP, colored by resolution. **e,** Gold-standard Fourier shell correlation curves (FSCs) for UV-DDB bound to the native NCP after polishing.

	Cryo-EM structure of native NCP-UV-DDB complex (EMDB-61242)	Cryo-EM structure of NCP-UV-DDB complex containing CPD (EMDB-61243) (PDB 9J8W)	Cryo-EM structure of UV-DDB bound to native NCP at SHL+/-2 (EMDB-61246)	Cryo-EM structure of UV-DDB bound to native NCP at SHL+/-3 (EMDB-61247)	Cryo-EM structure of UV-DDB bound to native NCP at SHL+/-6 (EMDB-61248)
**Data collection and processing**					
Magnification	81,000x	81,000x	81,000x	81,000x	81,000x
Voltage (kV)	300	300	300	300	300
Electron exposure (e–/Å^2)	58.529	61.159	58.6745	58.6745	58.6745
Defocus range (μm)	-2.5 to -1.0	-2.5 to -1.0	-2.5 to -1.0	-2.5 to -1.0	-2.5 to -1.0
Pixel size (Å)	1.06	1.06	1.06	1.06	1.06
Symmetry imposed	C1	C1	C1	C1	C1
Initial particle images (no.)	9,885,532	7,807,309	20,793,804	20,793,804	20,793,804
Final particle images (no.)	7,339	56,758	9,220	15,543	21,011
Map resolution (Å)	12.3097	3.37699	3.77822	3.85455	3.6
FSC threshold	0.143	0.143	0.143	0.143	0.143
Map resolution range (Å)	11.9669 to 24.2049	3.36029 to 5.71923	3.61821 to 10.8419	3.78751 to 17.1423	3.46987 to 10.8248
**Refinement**					
Initial model used (PDB code)		6R8Y, 6R8Z, 5B24			
Model resolution (Å)		3.37699			
FSC threshold		0.143			
Model resolution range (Å)		3.36029 to 5.71923			
Map sharpening *B* factor (Å^2)	-17.4719	-66.3652	11.0936	2.67195	-1.00939
Model composition					
Non-hydrogen atoms		27,958			
Protein residues		1159			
Nucleotides		289			
B factors (Å^2)					
Protein		79.31			
DNA		95.12			
R.m.s. deviations					
Bond lengths (Å)		0.005			
Bond angles (°)		0.742			
Validation					
MolProbity score		1.93			
Clashscore		9.32			
Poor rotamers (%)		0.82			
Ramachandran plot					
Favored (%)		93.34			
Allowed (%)		6.66			
Disallowed (%)		0			
**Model-to-data fit**					
CC mask		0.75			
CC box		0.74			
CC peaks		0.70			
CC volume		0.76			

Supplementary Table 1: Cryo-EM data collection, refinement and validation statistics
