# Supplementary material for: Structural basis of cyclobutane pyrimidine dimer recognition by UV-DDB in the nucleosome
Source: Nat Commun. 2025 Nov 11;16:9709. doi: 10.1038/s41467-025-65486-5 (PMC12606344; doi:10.1038/s41467-025-65486-5)
Supplement: Supplementary file 2 — Description of Additional Supplementary Files [file 41467_2025_65486_MOESM2_ESM.pdf]

## Description of Additional Supplementary Files

**Supplementary Data 1: Uncropped gels used in main and supplementary figures**

All uncropped gels used in this study are presented here. For clarity, the target bands were cropped and highlighted in the main and extended figures. All agarose gels, native-PAGE gels, and SDS-PAGE gels were captured using an Amersham Imager or a Typhoon Imager, and the resulting TIFF files were stored on HDDs or SSDs. These TIFF files were subsequently processed using Photoshop software, with contrast adjustments applied to improve visualization.
